# The Influence of Process Parameters in Radial Ring Rolling on Steel Ring Ovalization

**DOI:** 10.3390/ma19030484

**Published:** 2026-01-26

**Authors:** Piotr Surdacki, Andrzej Piotr Gontarz

**Affiliations:** Mechanical Engineering Faculty, Lublin University of Technology, 36 Nadbystrzycka Str., 20-618 Lublin, Poland; a.gontarz@pollub.pl

**Keywords:** ring rolling process, ovalization of rings, feed speed of the main roll, charge temperature, position of the calibration roll

## Abstract

Rolling steel rings is a key manufacturing process for producing components with high strength and dimensional accuracy, used, among others, in the automotive, aerospace, and energy industries. The quality of the products depends on the process parameters that affect their mechanical and geometric properties. One significant quality issue is ovalization, i.e., deviation from the ideal circular shape, which can complicate further processing or assembly. Therefore, analyzing the influence of rolling parameters on ovalization is crucial for ensuring high product quality and minimizing material losses. The aim of the research presented in this article was to determine the influence of the most important parameters of the ring rolling process—namely, billet temperature, forming tool speed, and the position of the calibrating rollers—on the ovalization of the rings produced. The results indicate that, among the parameters studied, the position of the calibrating roller engaged by the rolled ring has the greatest impact on ovality. Ovalization of the forging decreases with an increase in feed speed and a decrease in billet temperature. Higher feed speeds provide a more stable rolling process, which promotes the achievement of a more circular ring geometry. Lower billet temperatures are associated with better material strength properties, making it less susceptible to deformation under inertial forces compared to higher initial billet temperatures. The study of the influence of calibrating roller positions on ovalization showed that it is possible to determine an optimal configuration in which deviation from the ideal circular shape is minimized. Determining the optimal process parameters allows for producing components without the need for large-dimensional tolerances. Based on the results obtained, conclusions were formulated regarding the influence of the investigated process parameters on the ovalization of the finished ring.

## 1. Introduction

The hot ring rolling process is a widely used manufacturing technology for producing high-strength components with complex geometries, such as bearing races, gears, turbine rims, and aircraft structural elements. The main advantage of this method is its ability to produce components with high mechanical quality and increased resistance to cracking (compared to rings produced using other technologies), a favorable fiber structure, while simultaneously reducing the number of required manufacturing operations [1,2,3]. This process, characterized by the simultaneous reduction of wall thickness and an increase in ring diameter, offers significant advantages, including improved mechanical properties, material savings, and cost reduction compared to alternative manufacturing methods. Nevertheless, the complexity of the dynamic interactions between the rolls, mandrel, and the material being rolled makes maintaining the high geometric quality of the final product a significant challenge [1].

**Figure 1:**
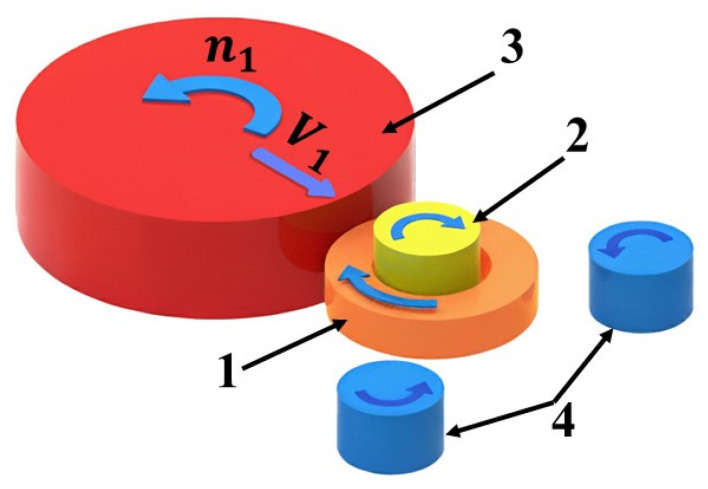
Schematic representation of the radial ring rolling process: 1—ring, 2—mandrel, 3—main roll, 4—calibration rolls.

The radial ring rolling process (Figure 1) involves the reduction of the cross-sectional area of the workpiece in the radial direction. The ring-shaped preform 1 is placed on the mandrel 2. The cross-section of the ring is reduced by the feed of the main roll 3, which moves with a linear velocity *V*_1_ toward the mandrel and rotates with an angular velocity *n*_1_, causing continuous rotation of both the ring and the mandrel around their own axes. Rolling mills are typically equipped with calibration rolls 4 to support and calibrate the workpiece [4,5,6].

One of the key challenges in ring rolling technology is ensuring adequate geometric accuracy [7], particularly maintaining circularity [8,9,10]. Deviations from the ideal circular shape—referred to as ovalization—can lead to difficulties in subsequent finishing operations, reduced functional properties of the component, and increased production costs [11]. The phenomenon of ovalization, also referred to as out-of-roundness, is the result of a complex interaction of multiple process factors, such as billet geometry [9,12,13,14,15], forming speed [9,10,11,15,16,17], rolling strategy [6,11,14], material properties [9,16], rolling force [4,11,18], temperature [16], and the stiffness of the tool system [4,12,14].

Previous studies have focused both on the analysis of the ring rolling process itself and on the application of simulation methods (including finite element—based approaches) to predict changes in geometry and stress states [4,9]. Research has focused on various aspects of the ring rolling process, analyzing the influence of factors such as tool feed rate [10,11,15], main roll rotational speed [11,15], guiding roll forces [9,11], and roll geometry [9] on ovalization, as well as defects such as “fish-tail” [4,15] and polygonal shape defects [11,19]. Despite the growing number of scientific studies, there is a lack of a comprehensive approach that would allow for a clear determination of the influence of individual process parameters on the extent of ovalization and enable its deliberate minimization at the process design stage.

Scientific publications describing the influence of process parameters on the dimensional accuracy of rings can be divided into two main groups depending on the rolling method, i.e., studies concerning radial–axial ring rolling (with conical rolls shaping the ring height) and radial ring rolling, which is the subject of this paper. The vast majority of publications address the radial–axial ring rolling process.

Lee and Kim [15], during their investigation of the radial–axial ring rolling process, found that increasing the ring diameter growth rate leads to a reduction in ovality, whereas too low a growth rate results in process instability and significant ovality. They also observed that a higher mandrel feed reduces ovality; however, an excessively high feed rate causes a loss of rolling stability. The study also examined the selection and correlation of the axial feed values of the conical shaping rolls with the velocities of the other tools. It was concluded that tool speed synchronization, rather than the absolute feed value, is the key factor. The researchers also highlighted the influence of the mandrel and main roll geometry, noting that larger tool radii promote more gradual deformation, resulting in reduced ring ovality.

In [12], the authors concluded that a large reduction in ring thickness per revolution results in local plastic deformation and an increase in ovality (a conclusion contrary to that of [15]). They also pointed out the continuous need to monitor and maintain proper speed relationships among all tools. It was further determined that the stiffness of the rolling mill has a significant effect on ovality. The authors additionally emphasized the importance of proper movement of the support rolls; their absence or improper displacement during the process leads to increased ring ovality.

Xie et al. [10], during their investigation of the radial–axial ring rolling process, pointed out that an excessively high feed rate—particularly in the late stage of rolling—leads to a rapid increase in ovality. They concluded that a high feed rate is acceptable only at the beginning of the process and must be significantly reduced in the final stage. The authors observed that ovality increases with increasing ring diameter and decreasing wall thickness. The researchers proposed dividing the radial–axial ring rolling process into several stages: Initial stage (1)—low feed rate: ensures stable ring gripping; Rapid stage (2)—higher feed rate: rapid diameter growth while maintaining sufficient stability; Stable stage (3)—constant diameter growth rate: limits the development of ovality; Slow (final) stage (4)—reduced feed rate: crucial for ovality reduction. The study indicates that the absence of the rapid stage (2) or an excessively short slow stage (4) results in a significantly higher ring ovality. The authors also stated that variable or excessively high friction promotes ring ovality and eccentricity.

Zayadi et al. [11], while investigating the radial–axial ring rolling process, found that the mandrel feed speed affects ovality mainly when it is excessively high relative to the rotational speed of the main roll, whereas the rotational speed of the main roll has a secondary influence. The authors also concluded that in order to limit ovality, efforts should be made to maintain a low ratio of the mandrel feed speed to the main roll rotational speed, rather than adjusting these parameters independently.

Arthington et al. [12], in their studies of the radial–axial ring rolling process, found that an excessively low rotational speed of the main roll leads to irregular contact with the rolled ring, local “sticking” of the material, and consequently an increase in ovality. The authors also concluded that thick-walled rings are significantly more resistant to ovality and that both excessively high and excessively low friction promote ovality.

Dong et al. [17], in their study of the radial–axial rolling process of aluminum rings, found that the forming force has a significant impact on ring ovality, as it causes uneven deflection of the ring. The authors also noted that thinner rings are more prone to developing ovality.

There are significantly fewer studies focusing on the investigation of the radial ring rolling method.

Tian et al. [16], in their study of the shaped ring forming process in a die, found that the key process parameter is the wall thickness of the product. Rings with thin walls are more susceptible to ovality. They also observed that ovality increases with rising friction, as confirmed by finite element simulations (uneven strain distribution). The authors further noted that a larger mandrel diameter leads to asymmetrical deformation and increased ovality. In their study, they proposed forming the examined products using dies that strongly constrain the outer diameter of the ring. This approach practically eliminates ovality and allows the rolling of very thin rings. They also emphasized that material homogeneity has a significant impact on the process.

Cleaver and Allwood [9], in their studies of the ring rolling process, found that the greater the mandrel diameter relative to the roll, the higher the tendency of the product to ovality. They also observed that a large reduction in billet thickness per revolution and a small wall thickness of the product contribute to increased ovality. The authors further noted that for materials with lower strength, shape defects are more pronounced.

Nayak et al. [18], based on studies of the radial ring rolling process of titanium rings, found that an excessively high main roll feed rate causes the material to flow unevenly, leading to increased ovality. They also noted that a feed rate that is too low results in prolonged contact time, which similarly worsens ring geometry. The authors concluded that the rate of wall thickness reduction is a key factor affecting ovality. Additionally, they found that, during the formation of titanium rings, a higher initial billet temperature improves material plasticity, which reduces ovality.

Zhihao et al. [7], in their study of the radial ring rolling process, found that an excessively high rotational speed of the ring promotes increased ovality. They also noted that high rolling mill stiffness suppresses vibrations and stabilizes ring shape. The authors recommend minimizing rolling time, as a prolonged process leads to the accumulation of errors and an increase in ovality.

The literature review indicates that research on the dimensional accuracy of rolled rings considers a variety of process parameters, depending on the rolling method, rolling mill design, material grade, and other factors. Based on the analysis of the studies presented, it was found that there is a lack of research on the influence of one of the fundamental process parameters—the billet temperature—on the ovality of the rolled ring. Furthermore, very few studies address the radial rolling method with fixed calibrating rolls, i.e., rolls that do not move during the rolling process. There are no studies aimed at determining the effect of the position of these rolls on process outcome and product quality. On this basis, it was concluded that it is advisable to conduct research considering these factors. Accordingly, the research objective was formulated to determine the influence of billet temperature and the position of the calibrating rolls on the ovality of hot-rolled rings, in correlation with the main parameter, which is the speed of the forming tool. The analysis conducted allowed for the identification of relationships between the investigated process factors and ring shape deviations, as well as the formulation of technological recommendations aimed at reducing the risk of undesirable geometric deformations. Although some studies indicate a relationship between process parameters and ring non-circularity, a comprehensive analysis of the mutual dependencies and their influence on ovality remains an area requiring further in-depth research.

## 2. Research Methodology

**Figure 2:**
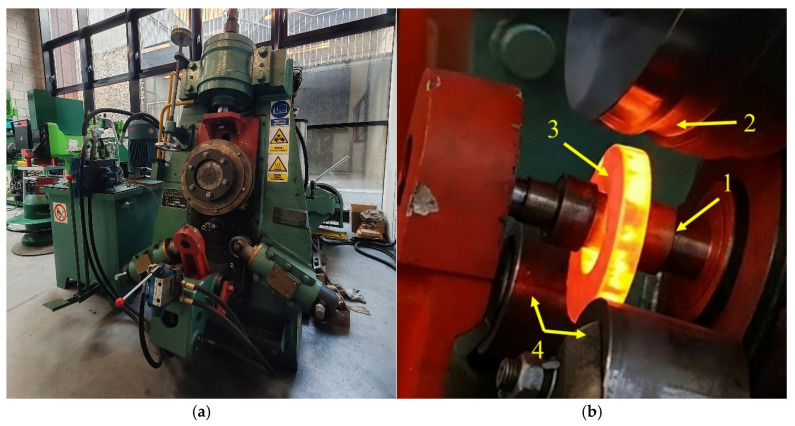
Ring rolling mill D51Y-160E used in the experiments (**a**) and the initial stage of the rolling process carried out using the following tools (**b**): 1—mandrel, 2—main roll, 3—ring, 4—calibration rolls.

Previous research by the authors has shown that the results obtained from simulations using various finite element method (FEM)-based software exhibit significant discrepancies compared to experimental results, particularly with respect to the shape of the rolled ring [5]. The results of the analysis for the three programs showed that the values of the diameters and heights of the ring obtained during the FEM analysis differed from the actual values by 7.45%, 9.95%, and 20.81%. This result is due to the number of simplifications used in the programs. The results demonstrate that—irrespective of the simulation software applied—axial metal flow increases and circumferential metal flow is lower than those obtained in the experiments. For this reason, an investigation of the influence of individual process parameters on the ovality value was carried out exclusively using experimental methods. The experiments were conducted using a commercial D51Y-160E ring rolling mill (Manufacturer: Wuxi Daqiao, Wuxi, China. The rolling mill is located at the Lublin University of Technology, Lublin, Poland) (Figure 2a) with the following parameters: maximum speed of main shaft—92 rpm, maximum roller linear velocity—45 mm/s, main motor power—30 kW, hydraulic motor power—7.5 kW. The beginning of the process with the hot workpiece placed in the rolling groove is shown in Figure 2b.

**Figure 3:**
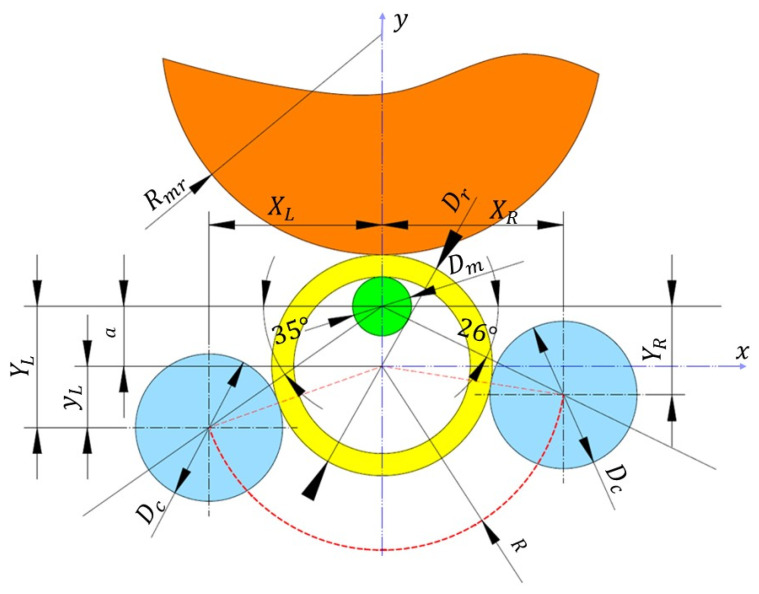
Geometric scheme of the idealized process at the final stage of rolling for the ring rolling mill used in the study; *R_mr_*—main roll radius, *D_m_*—mandrel diameter, *D_r_*—ring diameter, *D_c_*—calibration roll diameter, *R*—the radius of the circle passing through the centers of symmetry of the calibration rollers, *a*—the distance between the center of symmetry of the mandrel and the ring, *Y_R_*—the vertical distance between the center of symmetry of the mandrel and the right calibration roll, *Y_L_*—the vertical distance between the center of symmetry of the mandrel and the left calibration roll, *X_R_*—the horizontal coordinate of the center of symmetry of the right calibration roll in the OXY coordinate system, *X_L_*—the horizontal coordinate of the center of symmetry of the left calibration roll in the OXY coordinate system, *y_L_*—the vertical distance between the center of the ring and the left calibration roller.

Figure 3 presents a schematic illustration of the tool arrangement at the final stage of the ring rolling process. In the applied ring rolling mill, the working components have the following dimensions: main roll diameter *D_mr_* = 380 mm (radius *R_mr_* = 190 mm), mandrel diameter *D_m_* = 40 mm, and guide roll diameter *D_c_* = 100 mm. The rolls can be adjusted along the axis connecting their center of symmetry with the mandrel center, which is inclined to the horizontal plane at an angle of 26° (right roll) and 35° (left roll). These angles are always the same, regardless of other factors, and result from the design of the rolling mill. The calibrating rollers can only move linearly along directions inclined to the horizontal at specified angles (26° and 35°).

The tests were carried out on ring-shaped workpieces with the following dimensions: outer diameter 110 mm, inner diameter 50 mm, and height 20 mm. The rings were made of C45 steel (1.0503) with the chemical composition shown in Table 1. The workpieces were rolled into rings with the following dimensions: outer diameter 140 mm, inner diameter 114 mm, and height 22 mm. All tests were repeated at least three times.

The experiments were conducted using the following process parameters:

- main roll feed speed *V*_1_ = 5 mm/s, 10 mm/s, 15 mm/s, 20 mm/s;
- constant main roll rotational speed *n*_1_ = 60 rpm;
- workpiece heating temperature *T_w_* = 900 °C, 1000 °C, 1100 °C, 1200 °C;
- position of the right calibration roll *X_R_* = 110 mm, 115 mm, 120 mm, 125 mm, 130 mm, 135 mm, 145 mm (Figure 3);
- distance between the center of symmetry of the mandrel and the ring a = 37 mm.

It should be noted that in the rolling process, the calibrating roller plays a significant role; it is located on the side of the rolling tools, toward which the material moves after exiting the rolling groove. In other words, only the calibrating roller engaged by the rolled ring affects the process. In the analyzed case, this is the right-hand roller (see Figure 1). Therefore, only the position of this roller was studied. The left-hand roller does not participate in the rolling process; it only serves as a support point in the final phase of the process, when the ring has already reached its final dimensions. For this reason, the influence of the position of the left-hand roller on the process was not investigated.

Before rolling, the samples were heated in an electric resistance furnace to the required temperature for *t* = 20 min. Then, the workpieces were rolled at the required speed until the cross-sectional width (the distance between the shaping surfaces of the main roll and the mandrel) reached *W_f_* = 13 mm. After the main roll movement was completed (*V*_1_ = 0), the rotational motion was continued to ensure calibration of the ring along its entire circumference.

To describe ovality, the roundness deviation was determined, which measures the discrepancy between the actual contour of the ring and the ideal circular shape. After rolling, the outer diameter of each forged ring was measured in 16 planes. The ring was rotated by an angle of 22.5°. The first measurement was performed in the plane where the outer diameter of the ring was the largest. This plane can be most easily determined by rotating the ring between the two measuring arms (the greatest separation of the arms indicates the largest diameter). Starting from this plane, subsequent measurements were carried out in planes spaced at 22.5° intervals. In this way, the maximum and minimum outer diameters of the ring formed were determined. The roundness deviation (ovality) was defined as the difference between the maximum and minimum outer diameters of the ring relative to the nominal diameter (the diameter the ring would have without ovality), expressed as a percentage [20]:

(1) $$O=\frac{D_{f,max}-D_{f,min}}{D_{f,id}}\times 100\%,$$

where

*D_f,max_*—maximum value of the final outer diameter of the forged ring, mm;

*D_f,min_*—minimum value of the final outer diameter of the forged ring, mm;

*D_f,id_*—ideal diameter of the ring, mm.

## 3. Results and Analysis

### 3.1. Influence of Tool Velocity and Workpiece Temperature on Ovalization

**Figure 4:**
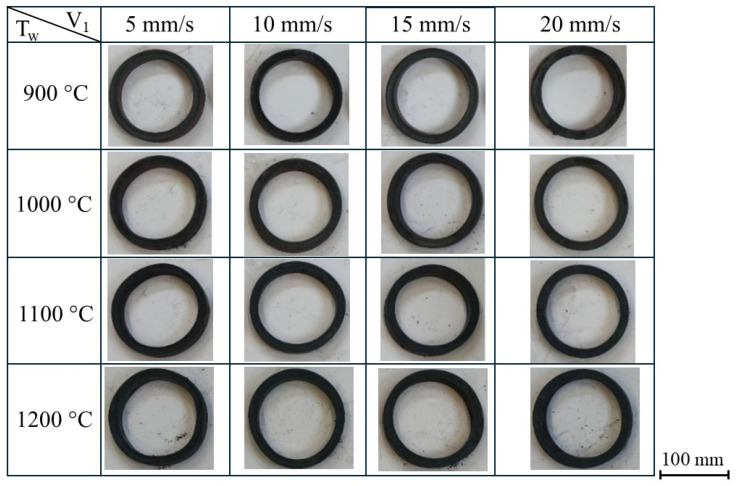
Rings obtained at different billet temperatures and main roll feed rates.

Figure 4 shows the rings produced during the experimental tests aimed at determining the influence of the main roll feed speed and the initial temperature of the ring on the ovalization of the ring. The experiments were conducted for the previously defined billet heating temperatures and feed rates *V*_1_ while maintaining a constant rotational speed of the main roll at *n*_1_ = 60 rpm. The center of the right calibration roll was located at a horizontal distance of *X_R_* = 120 mm from the center of the mandrel axis.

A visual examination of the ring geometry (Figure 4) reveals that as the feed rate of the main roll increases, the ring geometry approaches a more circular shape. This effect results from the forming time and the deformation characteristics of the ring rolling process. At low tool feed rates (*V*_1_ = 5 mm/s and 10 mm/s), the forming process exhibited ring instability—the ring was displaced along the mandrel and experienced vibrations due to the small thickness reduction per revolution of the main roll. At a higher feed speed, the forming process exhibited greater stability.

**Figure 5:**
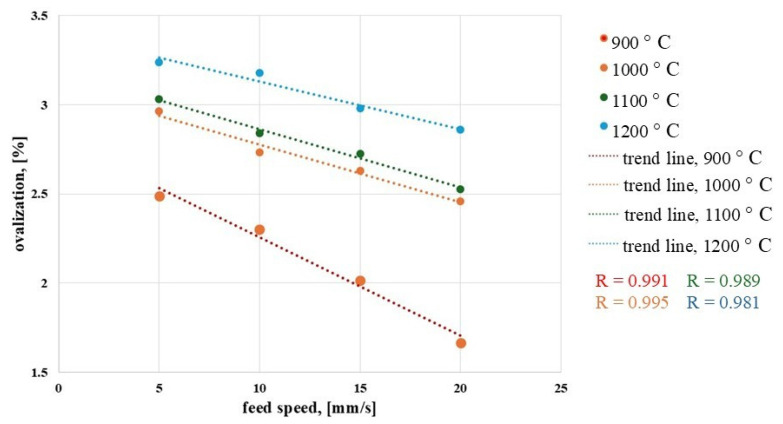
Dependence of ovalization on the main roll feed speed at different initial billet temperatures.

Figure 5 shows the ovalization values obtained for various main roll feed rates *V*_1_ and different billet heating temperatures.

For each trend line, R—the coefficient of the trend line—was determined. For each line, the values were as follows: *T_w_* = 900 °C—0.991, *T_w_* = 1000 °C—0.989, *T_w_* = 1100 °C—0.995, *T_w_* = 1200 °C—0.981. The values of the R coefficients in each case are close to 1, which indicates a good fit of the trend line with the experimental data. It can be observed that the value of the parameter analyzed decreases with increasing tool feed rate. This indicates that rings produced at higher feed rates have lower circularity deviations, resulting in a geometry more closely approximating a circle without defects. This observation justifies the application of higher feed rates for the shaping tool.

When analyzing the influence of the initial billet temperature on ovalization, it can be concluded that higher initial temperatures markedly worsen the geometry of the finished ring, which is evident even from visual evaluation (Figure 4). Elevated preform temperatures decrease the material’s mechanical strength, making it prone to the effects of inertial forces during rolling, which leads to greater shape deviations. In view of the ovalization effect, it is advisable to employ lower initial billet temperatures.

### 3.2. Position of Calibration Rolls

In the final stage of the rolling process, i.e., when the ring has already reached its final dimensions, its outer surface is supported at three contact points—by the main roll, the right calibration roll, and the left calibration roll. Assuming that the origin of the OXY coordinate system is located at the center of symmetry of the rolled ring (as shown in Figure 3), the coordinates of the centers of symmetry of the calibration rolls satisfy the equation of a circle with radius R.

For example, for the right calibration roll, this equation takes the following form:

(2) $${\left(Y_{R}-a\right)}^{2}+X_{R}^{2}=R^{2},$$

where

*Y_R_*—the vertical distance between the center of symmetry of the mandrel and the right calibration roll;

*a*—the distance between the center of symmetry of the mandrel and the ring;

*X_R_*—the horizontal coordinate of the center of symmetry of the right calibration roll in the OXY coordinate system.

(3) $$Y_{R}=X_{R}\;tg26{}^{\circ},$$

Thus, by substituting relation (2) into (3), we obtain a quadratic equation with a single unknown:

(4) $${\left(X_{R}\;tg26{}^{\circ}-a\right)}^{2}+X_{R}^{2}=R^{2},$$

Taking into account the position of the roll on the right (positive) side of the Y-axis, the solution of this equation is

(5) $$X_{R}=\frac{2\;a\;tg26^{\circ }+\sqrt{4\;a^{2}\;{tg26^{\circ }}^{2}-4({tg26^{\circ }}^{2}+1)(a^{2}-R^{2})}}{2{\;(tg26}^{2}+1)}$$

Similarly, the position of the left roll *X_L_* can be calculated by assuming an angle of 35° and taking into account its location on the left (negative) side of the Y-axis.

Formulas (2)–(5) describe the position of the rolls in a quasi-static process, that is, an idealized process occurring infinitely slowly, allowing the neglect of inertial forces acting on the ring. This represents an idealized situation (which does not occur in reality) and serves as a baseline state against which the positions of the right roll subjected to testing can be compared.

**Figure 6:**
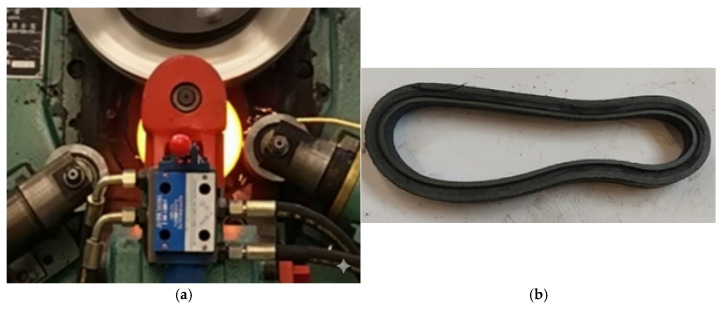
Ring rolling: (**a**) moment of contact between the rolled ring and the right calibration roll; (**b**) defective ring resulting from impact with the left calibration roll.

However, positioning the calibration rolls, especially the right one, at distances calculated based on Equation (5) does not guarantee optimal implementation of the process in terms of ring ovalization. In reality, the ring is not positioned symmetrically with respect to the axis of the mandrel and the main roll; rather, it deflects in the appropriate direction (in the studied case, to the right) in accordance with the rotational movement of the forming tool (Figure 6a). When the rolled ring reaches the appropriate diameter, it comes into contact with the right roll, which provides a support point and stabilizes its position. Based on the study, it was found that the position of the right roll is crucial for the proper course of the process.

The left roll comes into contact with the ring only in the final phase of the process. At the moment this roll touches the ring, the process should be completed. The outer surface of the ring contacts the tools at three points (the main roll and the two calibration rolls), and any further increase in the ring’s diameter leads to it being pulled between the main roll and the right calibration roll. It becomes deformed (Figure 6b), and the need to remove it from the machine causes production stoppage. For this reason, the left roll is sometimes used to halt the process, via a rotation sensor mounted on its axis. Upon contact, the ring sets the roll and the sensor into rotational motion, triggering a signal that stops the operational movement of the tools. In summary, it should be noted that the position of the right roll has a significant impact on the process, including on the ovalization of the ring.

**Figure 7:**
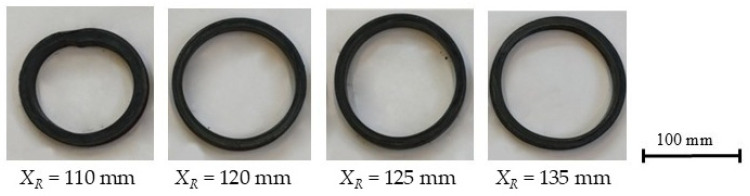
Selected rings obtained during the process carried out with a feed rate of 20 mm/s.

**Figure 8:**
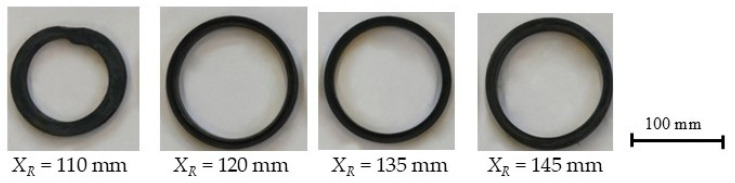
Selected rings obtained during the process carried out with a feed rate of 5 mm/s.

To analyze the effect of the position of the right calibration roll on the ovalization of the ring, the billets were initially heated to *T_w_* = 900 °C, a temperature at which rings with minimal shape error had previously been obtained. During the analysis, the position of the right calibration roll *X_R_* was varied. The analysis was carried out for two main roll feed rates, *V*_1_ = 5 mm/s and 20 mm/s, with a constant roll rotational speed of 60 rpm. For the lower feed rate, the study was conducted with the right calibration roll position *X_R_* ranging from 110 mm to 145 mm in 5 mm increments, whereas for the higher main roll feed rate, the right roll position was varied from 110 mm to 135 mm, also in 5 mm increments. Selected rings produced under the corresponding process parameters are shown in Figure 7 and Figure 8.

It should be noted that in both analyzed variants with *X_R_* = 110 mm, a proper ring was not obtained (Figure 7 and Figure 8). The roll was positioned too close to the mandrel axis, which caused slippage and deformation of the ring. For the remaining positions of the right calibration roll, proper rings were obtained.

**Figure 9:**
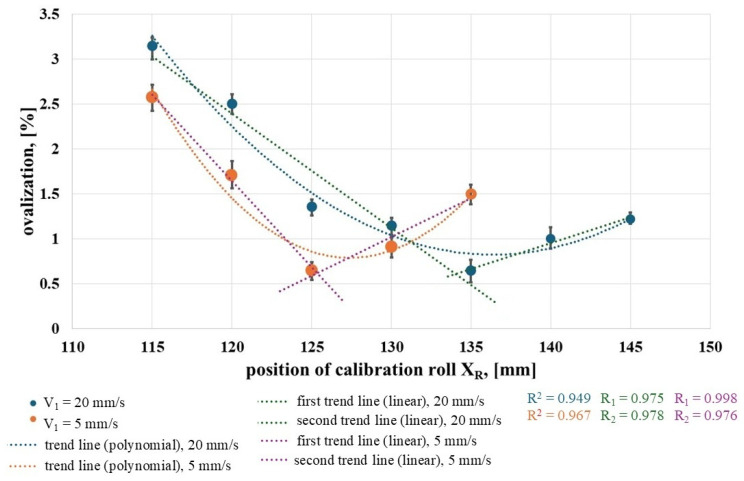
Relationship between ovalization and the position of the calibration roll for different main roll feed speeds.

Figure 9 shows the ovality value as a function of the right roll position *X_R_* for the two speeds analyzed. As can be seen, in both cases, the relationship has a parabolic shape with a distinct minimum, which indicates that there is a specific position of the right calibration roll (*X_R_* value) at which the ovality is minimal.

For each trend curves, *R*^2^—the coefficient of the trend curve—was determined. For each curve, the values were as follows: *V*_1_ = 5 mm/s—0.967, *V*_1_ = 20 mm/s—0.949. It should therefore be concluded that the models are reliable for describing relationships and making forecasts. A comparison of the *R* values of parabolic fits with two-stage linear fits was also undertaken. Cutting these two lines will allow you to better locate the minimum ovalization. Thus, for a speed of *V*_1_ = 5 mm/s, two fits with *R*_1_ = 0.975 and *R*_2_ = 0.978 were obtained. For *V*_1_ = 20 mm/s, *R*_1_ = 0.998 and *R*_2_ = 0.976 were obtained. For the main roll feed speed of *V*_1_ = 5 mm/s, the best ring shape was obtained at the right calibration roll position of *X_R_* = 125 mm, whereas for the feed speed of *V*_1_ = 20 mm/s, the optimal position was *X_R_* = 135 mm. As can be seen, smaller or larger values of the right calibration roll position result in greater ovality of the ring. Based on the results obtained, it can be concluded that, depending on the rolling process speed parameters, there is an optimal position of the right calibration roll at which the roundness deviation of the ring is minimal. It should be added that the optimal position of the right calibration roll (the horizontal distance between the roll axis and the mandrel axis) increases with an increase in the main roll feed speed *V*_1_ (at a constant rotational speed). It should also be noted that the effect of the right calibration roll position on ovality is greater than the effect of the roll feed speed and temperature on this shape parameter. Within the range of the investigated parameters, depending on the roll position, ovality ranges from 0.6% to 3.1% for *V*_1_ = 5 mm/s and from 0.6% to 2.5% for *V*_1_ = 20 mm/s.

## 4. Conclusions

Based on the results of the hot radial rolling process of rings made of C45 steel (1.0503), the following conclusions were formulated:

1. Among the investigated parameters (temperature, main roll feed speed, and right calibration roll position), the roll position has the greatest influence on ovality.
2. For a given main roll feed speed, it is possible to determine the optimal position of the right calibration roll that ensures minimal ovality of the rolled ring. For higher main roll feed speeds, the optimal roll position is closer to the shaping tools (the main roll and the mandrel).
3. With an increase in the main roll feed speed, a decrease in the ring’s ovality is observed. In addition, higher feed speeds provide a more stable rolling process, which promotes the achievement of a more circular ring geometry.
4. With an increase in the initial billet temperature, the ovality of the ring increases. This increase results from a reduction in the material’s strength properties, which makes it more susceptible to deformation under inertia forces. Therefore, it is recommended to carry out the ring rolling process at lower billet heating temperatures (900–1000 °C).
5. In none of the analyzed cases was perfect roundness achieved, which confirms the necessity of an additional calibration stage after rolling or the use of significant machining allowances.

## Figures and Tables

**Table 1:** Chemical composition of C45 (1.0503) grade steel [% wt].

C	Mn	Si	P	S	Cr	Ni	Mo	W	V	Al	Cu
0.42–0.5	0.5–0.8	0.1–0.4	max 0.04	max 0.04	max 0.3	max 0.3	max 0.1	-	-	-	max 0.3

## Data Availability

The original contributions presented in this study are included in the article. Further inquiries can be directed to the corresponding author.

## References

[1] Allwood J.M., Tekkaya A.E., Stanistreet T.F. (2016). The Development of Ring Rolling Technology. Steel Res. Int..

[2] Eruc E., Rajiv S. (1992). A summary of ring rolling technology–I. Recent trends in machines, processes and production lines. Int. J. Mach. Tool Manuf..

[3] Eruc E., Rajiv S. (1992). A summary of ring rolling technology–II. Recent trends in process modeling, simulation, planning, and control. Int. J. Mach. Tool Manuf..

[4] Gontarz A., Surdacki P., Michalczyk J. (2024). Research the Dimensional Accuracy of C45 Steel Ring Forgings Produced by Radial Rolling. Materials.

[5] Surdacki P., Gontarz A., Winiarski G., Samołyk G. (2021). Research of the Tool Velocity and Product Shape Aspects of the Hot Radial Rolling of C45 Steel Ring. Adv. Sci. Technol. Res. J..

[6] Surdacki P., Gontarz A., Winiarski G., Wójcik Ł., Wiewiórowska S. (2022). Research of Speed Parameters of the Ring Rolling Process. Adv. Sci. Technol. Res. J..

[7] Zhihao X., Zhijiang Z., Han L., Libo P. (2022). Research on dynamic measurement of hot ring rolling dimension based on machine vision. IFAC-Papers OnLine.

[8] Arthington M.R., Cleaver C., Allwood J., Duncan S. Measurement and control of variable geometry during ring rolling. Proceedings of the IEEE Conference on Control Applications (CCA).

[9] Cleaver C.J., Allwood J.M. (2019). Curvature development in ring rolling. J. Mater. Process. Technol..

[10] Xie D., Ouyang Q.Y., He L.Y., Xu W.J. (2023). Feed Curves for Controlling Ring Rolling Stability in Large-Scale Flat Ring Rolling Process. Materials.

[11] Zayadi H., Parvizi A., Farahmand H.R., Rahmatabadi D. (2021). Investigation of Ring Rolling Key Parameters for Decreasing Geometrical Ring Defects by 3D Finite Element and Experiments. Arab. J. Sci. Eng..

[12] Arthington M.R., Havinga J., Duncan S.J. (2020). Control of ring rolling with variable thickness and curvature. Int. J. Mater. Form..

[13] Cleaver C., Arthington M., Allwood J., Duncan S., Mortazavi S. (2016). Ring Rolling with Variable Wall Thickness. CIRP Ann..

[14] Hao T., Chen J., Zhang T., Qin Z., Wu Y. (2024). Study on Cavity Filling Defects and Tensile Properties of L-Shaped Profiled Rings. Materials.

[15] Kyung-Hun L., Byung-Min K. (2013). Advanced feasible forming condition for reducing rings preads in radial–axial ring rolling. Int. J. Mech. Sci..

[16] Duanyang T., Xinghui H., Lin H., Xuan H. (2022). An innovative constraining ring rolling process for manufacturing conical rings with thin sterna and high ribs. Chin. J. Aeronaut..

[17] Fang D., Tao Z., Yun-xin W., Lei L., Tie-wen H. (2023). Multi-scale simulation of flow behavior and microstructure evolution for AA2219 alloy during multi-pass ring rolling process. Trans. Nonferrous Met. Soc. China.

[18] Nayak S., Singh A.K., Gokhale H., Prasad M.J.N.V., Narasimhan K. (2023). Optimization of Ti-6Al-4V ring rolling process by FE simulation using RSM. Int. J. Mech. Sci..

[19] Moona H.K., Leeb M.C., Joun M.S. (2008). Predicting polygonal shaped defects during hot ring rolling using a rigid–viscoplastic finite element method. Int. J. Mech. Sci..

[20] Randhawa K. (2017). Ovality in Pipes. Int. J. Latest Technol. Eng. Manag. Appl. Sci..

